# Primary Malignant Tumours of Bone Following Previous Malignancy

**DOI:** 10.1155/2008/418697

**Published:** 2008-04-13

**Authors:** J. T. Patton, S. M. M. Sommerville, R. J. Grimer

**Affiliations:** The Royal Orthopaedic Hospital Oncology Service, Royal Orthopaedioc Hospital, Bristol Road South, Northfield, Birmingham B31 2AP, UK

## Abstract

Destructive bone lesions occurring in patients who have previously had a malignancy are generally assumed to be a metastasis from that malignancy. We reviewed 60 patients with a previous history of malignancy, who presented with a solitary bone lesion that was subsequently found to be a new and different primary sarcoma of bone. These second malignancies occurred in three distinct groups of patients: (1) patients with original tumours well known to be associated with second malignancies (5%); (2) patients whose second malignancies were likely to be due to the previous treatment of their primary malignancy (40%); (3) patients in whom there was no clearly defined association between malignancies (55%). The purpose of this study is to emphasise the necessity for caution in assuming the diagnosis of a metastasis when a solitary bone lesion is identified following a prior malignancy. Inappropriate biopsy and treatment of primary bone sarcomas compromises limb salvage surgery and can affect patient mortality.

## 1. INTRODUCTION

With the advances in the treatment of many cancers, patient survival has improved dramatically such that a second malignant neoplasm is now observed more frequently [1, 2]. It is well known that certain cancers are associated with an increased risk of a second cancer [3, 4], and that some treatments for cancer are in themselves known to be aetiological agents in the pathogenesis of a second cancer [5, 6] . It is commonly assumed that bone lesions occurring in patients who have already had a malignant neoplasm are metastases from that malignancy. If the lesions are multiple, this will almost always be true, but if the lesion is solitary then further investigation is necessary to exclude other pathologies such as another primary bone tumour.

## 2. MATERIALS AND METHODS

The patients for this study were identified from a prospectively recorded database. The records of 60 patients who presented to our institution between 1980 and 2001 with a primary bone tumour following a prior malignancy were reviewed. The data was analysed in three separate groups, although it should be pointed out that certain patients might belong to more than one group.

**Group 1**

Patients were categorised into this group if they had a primary malignant neoplasm that is known to have a strong relationship with the development of a secondary malignancy.

**Group 2**

This group included patients who had treatment of their primary tumour that is known to predispose to the development of secondary sarcoma. This includes all the radiation-induced sarcomas that arose within the radiation field.

**Group3**

Patients who developed a primary tumour of bone, subsequent to a different primary malignant neoplasm that was not clearly related to that neoplasm or to the treatment used for that neoplasm, were included in this group.

## 3. RESULTS

At latest follow up, 31 patients (52%) within the study group had died of disease relating to the primary bone sarcoma. The average time to development of metastases was 17.6 months (range 0–122 months).

**Group 1**

This group consisted of three patients (5%), all of whom had retinoblastoma as their first tumour. The sarcoma that subsequently developed was an osteosarcoma in two and a primary leiomyosarcoma of bone in the other. The ages at which the second tumour arose were ten, sixteen, and twenty three years, with a lag time from the original tumour of eight, fifteen, and twenty two years, respectively. All the second tumours were located in sites remote from the head and neck.

**Group 2**

There were twenty four patients (40%) who developed a sarcoma following previous radiotherapy for a primary tumour. Of these, twenty two were osteosarcoma and two were high-grade sarcoma with no identifiable differentiation. All of these secondary tumours were located in the previous radiation field. The average age of the patients at time of diagnosis of the second tumour was 45 years (range 16–80 years) with a lag time from the initial tumour of 14 years (range 4–31 years).

**Group 3**

Thirty three patients (55%) developed a primary malignant bone tumour following a previous malignancy that was not felt to be clearly related to or associated with the treatment of the first malignancy (Table 1). The average age at diagnosis of the second malignancy was 52 years (range 8–84 years) and the lag time between tumours was 5 years (range 0–18 years). The most common initial malignancy was carcinoma of the breast (thirteen patients), which was associated with chondrosarcoma in six cases. Three patients presenting with breast cancer developed bone sarcomas in the proximal limb girdle. Only one of these patients received radiotherapy as treatment for their breast cancer. The bone sarcoma in this patient occurred in the contralateral proximal humerus. Chondrosarcoma was the most common second malignancy, affecting twelve patients. The tumours occurred at a wide distribution of sites, most frequently in the proximal femur (eight patients). Three of the 33 patients presented with a soft tissue sarcoma as the initial malignancy. The subsequent primary bone tumours were located in sites remote from the initial sarcoma and were felt to have histology unrelated to the initial tumour.

## 4. DISCUSSION

Significant progress has been made in the treatment of and survival from numerous malignant neoplasms in recent years. The improvement in patient survival has revealed previously unrecognised sequelae. The second malignant neoplasm is a well-described entity [1, 2]. It is becoming clearer that genetic factors play a major role in the aetiology of the second malignant neoplasm. These factors are already well established in patients with tumours such as retinoblastoma and Ewings sarcoma [4, 7]. In this study, we have reported six cases of breast carcinoma associated with chondrosarcoma. Recently, this association has been reported by others and a genetic trait postulated [8]. It has also been observed that cancer patients themselves are at greater risk of developing a subsequent malignancy than the general population [1].

Treatments for cancer have also been implicated in the aetiology of a second malignant neoplasm. Radiotherapy is a well-established causative agent and is dose dependent [6]. More recently, certain chemotherapeutic agents have also been implicated [9].

There is a tendency to assume that a solitary bone lesion following a malignancy is a metastasis from that malignancy. This is especially true if the patient is over 40 [10] , and the interval between the initial malignancy and the second presentation is short. It was unfortunately beyond the capability of this study to provide a denominator of the number of patients with metastatic solitary bone lesions from the same population that presented to our tertiary referral centre with second and different primary bone tumours.

It is sobering to see that for the Group 3, patients in this study the average interval between tumours was only five years, and the average age of the patients was 52 years. In patients who have had radiotherapy to a previous lesion (Group 2), the assumption that the subsequent bone lesion is recurrent disease from the first malignancy can result in incorrect or substandard therapy.

The potential deleterious effects of a poorly performed biopsy have been well described by Mankin et al. [11] . There are now several reports illustrating the disastrous effects of inappropriate treatment of primary bone sarcomas, usually brought about by the assumption that the lesion is a metastasis [12]. In this study, patients managed in our tertiary referral centre had a biopsy prior to initial treatment. However, we know of at least 3 cases where presumption of the diagnosis of metastasis led to substandard treatment. This was caused by the assumption that the lesion was a metastasis, and the failure by the general orthopaedic surgeon to maintain an index of suspicion that a solitary bone lesion could be a primary bone sarcoma. Whist no particular traits were established, lesions in atypical sites (e.g., scapula, fibula) should be regarded with extrasuspicion. Most of the lesions in this study had characteristic appearances of primary bone sarcomas rather than metastases.

Internal fixation of these lesions can spread tumour cells along the whole length of the bone, rendering limb salvage surgery impossible. Clearly, therefore, we would urge caution in the treatment of solitary bone lesions, even in the presence of previous malignancy. There is rarely error in temporarily delaying potentially deleterious surgery or radiotherapy, imaging the patient appropriately, and performing a biopsy by a surgeon experienced in orthopaedic oncological procedure [13].

## Figures and Tables

**Table 1:** Data for the Group 3 patients, in whom there was no clearly defined association between malignancies.

Patient	Age (at diagnosis of second tumour)yrs	Sex	Second tumour (primary bone sarcoma)	Initial malignancy (Ca: carcinoma)	Site of second tumour	Lag (time to second tumour)yrs
1	72	F	Chondrosarcoma	Ca breast	Prox femur	14
2	59	F	Chondrosarcoma	Ca breast	Prox humerus	1
3	50	F	Chondrosarcoma	Ca breast	Prox femur	16
4	58	F	Chondrosarcoma	Ca breast	Prox humerus	2
5	60	F	Chondrosarcoma	Ca breast	Distal femur	0
6	60	F	Chondrosarcoma	Ca bronchus	Prox tibia	2
7	72	F	Chondrosarcoma	Ca colon	Distal femur	4
8	44	M	Chondrosarcoma	Ca oesophagus	Pelvis	6
9	58	M	Chondrosarcoma	Ca rectum	Prox tibia	7
10	82	M	Chondrosarcoma	Leukaemia	Prox femur	6
11	52	F	Chondrosarcoma	Malignant melanoma	Pelvis	3
12	66	F	Chondrosarcoma	Ca breast	Metatarsal	0
13	66	F	Chordoma	Ca breast	Sacrum	0
14	55	F	Chordoma	Ca breast	Sacrum	12
15	29	F	Ewings sarcoma	Ca kidney	Prox femur	0
16	12	F	Ewings sarcoma	Hodgkin's lymphoma	Fibula	6
17	9	M	Ewings sarcoma	Leukaemia	Prox tibia	5
18	78	M	Fibrosarcoma	Malignant fibrous histiocytoma (soft tisssue)	Pelvis	2
19	41	F	Fibrosarcoma	Malignant melanoma	Prox femur	3
20	84	F	Leiomyosarcoma	Ca breast	Distal fibula	8
21	47	F	Malignant fibrous histiocytoma	Hodgkin's lymphoma	Distal femur	6
22	39	M	Malignant fibrous histiocytoma	Seminoma	Distal femur	10
23	27	F	Malignant fibrous histiocytoma	Ca breast	Prox femur	2
24	66	M	Osteosarcoma	Ca bladder	Scapula	3
25	63	F	Osteosarcoma	Ca breast	Prox femur	5
26	10	M	Osteosarcoma	Hodgkin's lymphoma	Prox humerus	3
27	33	F	Osteosarcoma	Malignant melanoma	Prox tibia	4
28	8	F	Osteosarcoma	Rhabdomyosarcoma	Prox tibia	5
29	12	M	Osteosarcoma	Rhabdomyosarcoma	Prox femur	11
30	81	F	Sarcoma	Ca breast	Prox fibula	0
31	72	F	Sarcoma	Ca breast	Scapula	3
32	69	M	Spindle cell sarcoma	Ca prostate	Scapula	1
33	81	F	Spindle cell sarcoma	Ca colon	Prox tibia	18
